# Validation of a novel online depression symptom severity rating scale: the R8 Depression

**DOI:** 10.1186/s12955-020-01654-z

**Published:** 2021-06-12

**Authors:** Yuki Takao, Eduardo Figueroa, Kevin Fernand Jean Berna, Youjin Jo, Lee Andrew Kissane, Kimio Yoshimura, Richard Tranter, Richard J Porter

**Affiliations:** 1 Tower Hamlets Centre for Mental Health, Bancroft Rd, London, E1 4DG UK; 2 American Clinic Tokyo, Niikura Building 3F, 1-7-4 Akasaka, Minato, Tokyo 107-0052 Japan; 3 University of Luxembourg, 2, avenue de l’Université, 4365 Esch-sur-Alzette, Luxembourg; 4 Oregon State University, 1500 SW Jefferson St., Corvallis, OR 97331 USA; 5 Department of Health Policy and Management, Keio University School of Medicine, Tokyo, Japan; 6 Mid North Coast Local Health District Mental Health Services, Port Macquarie, NSW 2444 Australia; 7 Department of Psychological Medicine, University of Otago, Christchurch, New Zealand; 8 Tokyo Mental Health, 2-4-6 Shintomi, Chuo, Tokyo 104-0041 Japan

**Keywords:** Depression, Mood disorder, Outcome measure, Scale, Questionnaire, Validation, Patient-rated, Self-report, e-mental health

## Abstract

**Background:**

An automated web-based assessment and monitoring system (www.psynary.com) has been developed to assist non-specialist clinicians in managing common mood and anxiety disorders. Psynary promotes the use of standardised outcome measures to assess symptom severity and optimise treatments with the aim of improving outcomes and enabling faster recovery. This paper analyses the results from two parallel studies in New Zealand and Japan (OptiMA-1 NZ and Japan) to assess the validity of the R8 Depression scale, one of the system’s core outcome measures.

**Methods:**

Clinical samples were recruited from a public secondary care and a private psychiatry clinic. Participants completed the outcome measures for the study via the online Psynary system. The R8 Depression scale is a 30-item questionnaire which includes all symptom domains covered in the ICD-10 classification of depression. The Patient Health Questionnaire (PHQ-9) was completed at the same time points as the R8 Depression, with a smaller sample also completing a paper-based Quick Inventory of Depressive Symptomatology (QIDS-SR16). Internal validity was quantified via Cronbach’s alpha and Guttman lower bounds method. External validation against the PHQ-9 and QIDS used the Pearson’s and Kendall’s correlation coefficients. Severity categories were set using a multivariate regression model.

**Results:**

270 patients participated in the study and completed a maximum of 1 baseline and 5 reviews within a 90-day period, giving a total of 1124 assessments with the PHQ-9 also being completed in 1053 of these assessments. R8 Depression normative data was also collected from 204 non-clinical volunteers with 187 of these also completing the PHQ9. Internal reliability scores were all higher than 0.9 (n = 1328). There was overall good external validity when comparing the R8 Depression to the PHQ-9, with a correlation of 0.91 for the combined normative and clinical samples (n = 1240).

**Conclusions:**

The R8 Depression has been developed as a patient-rated outcome measure for depression for administration on an online system called “Psynary”. It has high internal and external validity against current widely used scales. Further work is underway to determine the sensitivity to change of the R8 Depression.

## Background

Depression is often a recurrent or chronic condition across the lifespan [1]. As well as the direct impact on health, depression has enormous direct and indirect costs for the individual, their families and society. Depression is the leading cause of sickness absence from work in industrialised regions such as Europe [2], and the annual economic burden in the US in 2010 estimated at $210 billion, 50% of which was workplace-related [3]. The prevalence of common mood and anxiety disorders in primary care far exceeds the availability of mental health specialists, and there is growing awareness of the need to look beyond the mental health workforce to meet treatment demands [4].

The goal of treatment for depression is complete remission from depressive symptoms. Achieving remission is crucial as residual depressive symptoms are the strongest predictor of early relapse and are strongly associated with poorer functional outcomes [5]. Achieving earlier remission from a depressive episode may be associated with reductions in the enormous indirect economic costs of the condition [4, 6]. However, STAR*D, the largest clinical trial examining outcomes for treatments of depression, highlighted that only a third of patients achieved remission on first-line treatments and up to four successive trials of different regimes were required to double the remission rate [7]. Unfortunately, clinicians often treat depression sub-optimally [8]. Where treatment is initiated, clinicians often wait until at least 6 weeks prior to attempting optimisation of dosages or changing medications, with patients sometimes left on ineffective or even harmful treatments for longer [9]. Optimising treatment may take many months, with the lost opportunity of potentially achieving earlier remission and functional recovery, and a failure to realise the potential indirect economic savings for society.

A way of improving the selection of modalities of treatment is by measuring symptom severity using standardised clinical measures [10]. Measuring detailed symptom outcomes enables detection of response to treatment as early as 1 week after initiation [11]. If a 20% improvement in depressive symptoms were not detected within 2 weeks from the start of the treatment, only 11% would respond to that treatment if it were maintained for longer [12]. Providing clear feedback to patients on their outcomes may also enhance treatment response [13]. Therefore, the routine use of detailed outcome measures offers the potential for prompt optimisation and personalisation of treatments for depression and faster functional recovery. Nevertheless, the adoption of such measures in busy clinics remains low [14]. This could be due to time constraints, limited awareness or knowledge of the clinician, and/or availability of the scales (including licensing restrictions, costs).

To address these barriers to translating insights from clinical trials into routine clinical practice, an international collaboration of specialist psychiatrists and IT engineers have developed an automated online assessment and outcome monitoring system (www.psynary.com), which enables routine collection of detailed patient-rated outcomes to accelerate the cycle of treatment optimisation [15]. Such a platform is readily scalable to extend specialist expertise to primary healthcare settings, particularly to support non-specialist clinicians to meet the enormous clinical need associated with common mood and anxiety disorders. The system has been developed and piloted in public and private specialist clinics in New Zealand and Japan. It is free to access and use.

This paper describes the outcomes from two parallel studies designed to validate the primary depression outcome measure, the R8 Depression, developed specifically for the Psynary system. This novel patient-rated outcome measure was designed to fulfil the following key requirements:

1. Items encompass symptoms commonly attributed to clinical domains of: mood; psychomotor changes; vegetative symptoms (sleep and appetite); cognitive symptoms (e.g. concentration and forgetfulness); and anxiety;
2. Items must be easy to understand for patients and there must be clear reference points for scoring each item;
3. Items must have clinical utility for clinicians and cover the range of clinical questions typically addressed when assessing depression severity;
4. Cut-offs for the total score must align with National Institute of Clinical Excellence (NICE) definitions of depression severity, enabling the use of this metric to stage interventions for depression in accordance with the NICE guidelines [10];
5. The total scores from the repeated completion of the novel questionnaire must be sensitive to the clinical effects of treatment and, therefore, must reliably detect a significant response to treatment at an early stage.

This study focuses on the internal validation, external validation and factor analysis of the R8 Depression scale.

## Methods

### Study design

OptiMA1-NZ and OptiMA1-Japan are parallel studies adopting the same methodology in New Zealand and Japan respectively to validate the primary outcome measures used by an online system, Psynary. This paper describes the validation of its primary depression outcome measure: the R8 Depression. Data from both OptiMA1-NZ and OptiMA1-Japan studies were combined for analysis in this paper. The two studies were approved by the clinical research ethics committees of University of Otago (New Zealand) and Asai Hifuka Institutional Review Board (Japan).

Participants were recruited from patients registered on the online Psynary system by the public community mental health clinic at Nelson Marlborough District Health Board (NMDHB) in New Zealand and by the private clinic serving the Tokyo English-speaking expat population, American Clinic Tokyo (ACT) in Japan. Patients with probable mood or anxiety disorders who registered to the online Psynary system between March 24th 2016 and October 25th 2018 were invited to complete either an online or written consent process prior to participating in the study. Inclusion criteria included completing psynary in the English language, being over 18 years of age for NZ, or 20 years of age for Japan, and having an ICD-10 (International Classification of Diseases) [16] diagnosis of a current depressive episode (unipolar or bipolar) or anxiety disorder (ICD-10 F31.3, F31.4, F31.81, F32.1, F32.2, F33.1, F33.2, F40-F43) confirmed by the treating clinician at their initial appointment.

As part of the Psynary assessments, participants were guided through and asked to complete the R8 Depression as well as the Patient Health Questionnaire (PHQ-9) [17]. A maximum of 6 assessments were included for each participant; one baseline assessment and up to five follow-up/review assessments if completed within 90 days from baseline. Review data were included to ensure the full range of depressive symptom severities was captured as patients progressed through their recovery.

The PHQ-9 was selected as the primary comparison measure for the external validation due to its widespread international use in primary care, which is the target clinical environment for use of Psynary. There were a priori concerns that the restricted number of items of the PHQ-9 may limit its sensitivity, so, where possible, Quick Inventory of Depressive Symptomatology (QIDS-SR16) questionnaires, which consists of 16 items, were also collected in a smaller sub-sample at the Tokyo clinic, thus providing a further opportunity to triangulate external validation between the questionnaires. Licensing restrictions prevented QIDS from being incorporated into the electronic platform.

The Psynary system is completely anonymous with patients being allocated a username (a colour and a number) on registration and a temporary password which they change after their first login. All patients complete a generic consent process when using Psynary for the first time. Additional consent forms for the OptiMA1 studies were completed. It is worth noting that Psynary does not collect any personal identifiable information. For instance, Psynary collects approximate age (to the nearest year) but does not collect the date of birth. Participating clinics keep their own records linking Psynary usernames with patient identification, which is held on their clinical information systems and not shared with Psynary.

The clinics using Psynary initiated and optimised treatments for mood and anxiety disorders based on established local and international guidelines [18]. Patients were encouraged to complete Psynary reviews every 1 to 2 weeks or prior to clinic appointments. The baseline assessment takes 40 min to complete on average while review assessments take 10 min.

In addition to the clinical sample, a normative sample was recruited, comprising friends, relatives and work colleagues of the international research teams. Personal e-mail invitations were cascaded as per the research ethics framework of the studies. A description of the study and a link to the online R8 Depression and PHQ-9 questionnaires were provided in the e-mail, which was entirely anonymous. In addition to the R8 Depression and PHQ-9, information regarding gender, age, nationality, first language and educational background was also collected for the normative sample. Finally, participants in the normative arm of the study were asked about current and past treatment for depression and family history of depression.

### Description of clinical metrics

The R8 Depression is a 30-item-questionnaire that covers all the symptom domains included in the ICD-10 classification of depression, as well as commonly associated symptoms, e.g. anxiety, and symptoms associated with melancholia and atypical depression. Each symptom item is scored on a 0 to 3 scale. For items covering appetite increase or reduction and weight gain or loss, the highest scores on either item are used. Therefore, 28 items are summed to give the total score, the maximum score being 84. To ease interpretation on Psynary the R8 Depression score is calculated as a percentage of this total score. The development of the R8 Depression is described below, and the questionnaire is reproduced in “Appendix”.

The PHQ-9 is a widely used international measure of depressive symptoms used to screen for depression and measure outcomes to treatment [19]. It is a 9-item-scale with each item rated from 0 to 3 and individual items summed to generate a total score. There are well-established thresholds for remission and mild, moderate, moderately severe and severe depression.

The QIDS-SR16 is a 16-item-scale developed from the larger 30-item Inventory of Depressive Symptomatology (IDS-SR30). It is a self-reported questionnaire which is also widely used in practice and has strong psychometric properties with appropriate sensitivity to change [20].

### Development of R8 Depression

The starting point for development of the R8 Depression was identifying a sufficient number of items to cover all the clinical domains included in the ICD-10 classification of depression: low mood (items 1 and 11); anhedonia (item 2); fatigue (item 6); poor concentration (item 28) and forgetfulness (item 10); reduced self-esteem/confidence; excessive guilt (item 8) and unworthiness (item 4); hopelessness (item 3); suicidal ideation (item 22); disturbed sleep; disturbed appetite. The somatic domain qualifier in ICD 10 equates to melancholic depression and includes symptoms of: loss of emotional reactivity, early morning awakening; psychomotor agitation (item 13) or retardation (item 9); weight loss (item 7); loss libido (item 15). Further items were then added to encompass important symptoms or problems routinely enquired about in specialist psychiatry clinics when assessing presentations of depression. For instance, specific reference to health anxiety (item 14) was included due to the prevalence of this symptom in depression, particularly amongst older patients [21]. Due to the prevalence of somatic symptoms as proxy-presentations for depression across many cultures item 20 refers to experience of unpleasant physical symptoms [22]. As anxiety symptoms are reported as occurring in up to 90% of patients presenting with depression [23], it is important to include an item relating to this (item 16). The identified subgroup of atypical depression tends to present with increased sleep and appetite, and hence the scale needed to determine both abnormal increases and decreases in vegetative symptoms and weight. Stemming from this, items relating to appetite (items 25 and 27) and weight (items 7 and 12) change were split into appetite reduction and increase, and weight loss and gain items. A logic operation was then introduced into the scoring of the R8 Depression to include only the highest rated of either of these pairs of items.

Due to sleep architecture being differentially affected in depression, it was important clinically to delineate falling asleep (item 30), sleep disturbance (item 26), early morning awakening (item 23), and increased sleep (item 18). This required four separate items. Aspects of social and daily functioning are commonly affected by depression and are an important focus of psychiatric assessment. Hence items of socialising (item 5), irritability (item 21), indecisiveness (item 29), motivation (item 24), daily activities (item 19), and sensitivity to criticism (item 17) have been included.

A four point Likert scale for items was already established as a standard amongst existing depression rating scales and it was decided to adhere to this standard. The anchor points are specifically defined for each item with the intent of mirroring the types of questioning used in psychiatric assessment. The extreme anchor points for each item were defined to indicate absence of that particular symptom or problem through to the most extreme clinical presentation. In this respect, it was important to draw upon clinical psychiatric expertise of the types of severe depression often requiring inpatient admissions and treatments such as Electroconvulsive Therapy (ECT). An example is the upper anchor point for the item on guilt which refers to delusions of guilt. One expectation of the R8 Depression was its ability to capture all gradations of severity of depression seen across the clinical spectrum from primary care to secondary care settings, without encountering a ceiling effect for the ratings.

The wording of all anchor points for items went through many reviews and revisions. There was an initial attempt to use plain English and avoid convoluted sentences, often seen in other scales. There were multiple reviews of wording by non-specialist, non-clinicians in a normative sample and patients.

In particular, previous work on translating another widely used depression rating scale [24] had identified the importance of subtle phrase variations in distinguishing between the absence of a symptom or problem and the threshold for indicating its mild occurrence (i.e. scoring between 0 and 1 on an item). Statistical analysis of responses in the initial normative population field testing revealed outlier items with significantly increased frequency of scores greater than 0 where subtle adjustments to the phrasing of the second anchor point had to be made.

The process of translating the R8 Depression into Japanese, a language that is very precise and specific, revealed ambiguities in the initial English phrasing for certain item anchor points. Considering international translation early in a clinical scale’s development is an important tool in further refining the clarity of item anchor points and the scale’s generalizability globally.

Valid criticisms have been raised against current depression outcome measures employing simple summation of symptom item ratings to generate a total severity score, arguing that different symptoms may contribute differentially to illness burden, that there is evidence of differential variation if symptoms change over time during recovery, and that current unitary models of depression are likely to conceal important sub-syndromes or entirely separate conditions [25, 26]. The Psynary platform will allow for the development of nuanced latent scoring approaches for the R8 Depression, that will map to important sub-syndromes, accurately and sensitively capture response to treatment and ultimately contribute to treatment response prediction. This is an expected stage of development once the database has reached a greater level of maturity. At this stage in the system development, however, it is important for the R8 Depression to reflect existing norms for outcome measures and to accurately map total scores onto existing definitions of remission and depression severity and hence to integrate within widely used clinical guidelines that used such severity categories to stage treatments for depression [10].

### Analysis

The distributions of the R8 Depression and PHQ-9 scores were calculated across the various samples, to assess the performance of these metrics across varying presentations of depression severity, particularly to identify potential ceiling or floor effects associated with the measures, and to evaluate the suitability of either parametric or non-parametric analyses.

The Cronbach’s alpha coefficient was calculated to assess the internal reliability for the completed assessments, which enabled direct comparison with other clinical measures that have been published, including depression questionnaires [27, 28]. Given the non-Gaussian data distributions when the baseline data was combined with the reviews and normative data, the Guttman’s lower bounds method [29] was also computed for further means of internal validation.

To explore the underlying factors of the R8 Depression and to understand the distribution of questions per factor, a conjunction of Principal Components Analysis (PCA) and Exploratory Factor Analysis (EFA) was implemented. Baseline data was used and included all variables except libido due to the low loadings in factors and lowering of the explained variance. Weight gain/weight loss and increased appetite/loss of appetite variables were reduced to one variable each called “weight change” and “appetite change”. PCA was first used to ascertain the validity of the component reduction procedure and to quantify the number of factors that are underlying in the data. Due to the highly correlated factors, the Direct Oblimin (Delta = 0) rotation method was applied, obtaining results that satisfy the assumption of sampling adequacy of the whole dataset via the Kaiser–Meyer–Olkin (KMO) test. To further ascertain the contents of these factors, EFA was run with the same parameters as the PCA. The Kaiser criterion of including factors with Eigenvalues greater than 1 was set as the method for determining factor solutions prior to the analysis.

While external reliability of the R8 Depression was assessed by calculating the Pearson’s product-moment correlation coefficients in line with most other depression rating scale validation studies, the non-normal distribution of the data set should preference the use of Kendall’s tau [30, 31]. Both these tests were used to examine correlations between: (a) R8 Depression scores and PHQ-9 scores; and (b) R8 Depression scores and QIDS scores. Tests were two-tailed and the p significance value was 0.05. The external validity was tested for three progressively larger datasets: baseline assessments only for the clinical sample, both baseline and review assessments for the clinical sample, and clinical (baseline and reviews) and normative samples. These datasets were expected to represent different distributions of depression severity. The baseline clinical sample was expected to represent the more severe range of depression. The baseline and review samples, i.e. the total clinical sample, was expected to include patients who had experienced various degrees of recovery and, therefore, to be skewed towards mild and moderate depression. The largest sample including the normative data was expected to show a distribution of depression symptom severity skewed more towards remission. These different samples were analysed to quantify the effect of varying distributions of depression severity on the scores of external validities. The QIDS sample enabled a triangulation to assess external validity between the R8 Depression, PHQ-9 and QIDS.

For the combined clinical and normative sample used to establish severity categories for the R8 Depression, there was sufficient improvement in the uniformity of the data for an assumption of normality to be fulfilled in regards to the use of the linear regression model. Several comparisons were made between the R8 Depression, PHQ-9 and QIDS with sub-samples represented as a linear regression equation.

All statistical analyses were performed using SPSS version 24 (IBM Corp. Released 2016. IBM SPSS Statistics for Windows, Version 24.0. Armonk, NY: IBM Corp.).

## Results

The study included data from 270 patients, 62 of which were from the New Zealand clinic and 208 participants from the Tokyo clinic. Data from 854 follow-up assessments were included, accounting for a total of 1124 clinical Psynary assessments. A total of 193 QIDS questionnaires were completed to assess a second further validation against current gold standard clinical measures.

A normative sample of 204 participants completed the R8 Depression and of these 187 also completed the PHQ-9.

### Sample characteristics

53.8% (n = 144) of the 270 patients were female. The mean age was 34.3 years and Table 1 shows the distribution of patients in different age groups (range 18–72 years). The mean duration of episode of mood or anxiety disorder prior to presentation to the clinics was 20.6 months. Nearly a third of patients (31.5%) had more than one treatment change prior to visiting the clinic. Their employment status is shown in Table 1, with a total of 70.4% reporting being in employment or self-employed (both part-time and full), which shows retainment of a reasonable degree of functioning amongst the sample.

**Table 1:** Patient characteristics (n = 270)

Patient characteristics	%	n
Clinic		
New Zealand	23	(62)
Japan	77	(208)
Gender		
Female	53.8	(144)
Age		
18–24	18.5	(50)
25–34	39.3	(106)
35–44	23.3	(63)
45–54	14.8	(40)
55+	4.1	(11)
Employment		
Employed full-time	49.3	(133)
Employed part time	11.1	(30)
Self-employed full-time	4.1	(11)
Self-employed part time	5.9	(16)
Homemaker	6.7	(18)
Student	13.3	(36)
Retired	0.7	(2)
Unemployed	7.8	(21)
Permanently sick	1.1	(3)

For the normative sample (n = 185), 71% of the patients were female, with a median age range of 30 to 39 years. 6% of the normative sample reported to be currently receiving treatment for depression, whilst 21% had a past history of treatment for depression and 46% had a family history of depression. This is expected given the prevalence of depression in the community [32].

The clinical diagnoses determined by the treating clinician are shown in Table 2. 34.9% were diagnosed with a moderate depressive episode and 52.6% were diagnosed with a severe depressive episode. Those who were determined not to have a depressive episode (7.8%) or mild episode (12.4%) had an anxiety disorder as their primary diagnosis.

**Table 2:** Clinician clinical diagnosis

Clinician clinical diagnosis	%	n
Depressive episode		
Mild	12.4	(31)
Moderate	34.9	(87)
Severe	52.6	(131)
None	7.8	(21)
		Total = 270
Pattern		
Single episode	24.8	(67)
Recurrent episode	50.4	(136)
Bipolar I	5.2	(14)
Bipolar II	17.0	(46)
Other	2.6	(7)
		Total = 270
Anxiety disorders		
Agoraphobia	16.3	(44)
Social phobia	9.3	(25)
Specific phobia	13.3	(36)
Panic disorder	21.8	(59)
Generalised anxiety disorder	13.0	(35)
Obsessive compulsive disorder	14.8	(40)
Post-traumatic stress disorder	4.1	(11)

For the 270 baseline clinical Psynary assessments completed, the mean R8 Depression score was 38.6 (± 16.7) (maximum score of 84) and the mean PHQ-9 score was 13.8 (± 7.7). Table 3 shows how the means of the total scores changed from baseline through consecutive reviews for both the R8 Depression and PHQ-9.

**Table 3:** Mean (S.D.) R8 Depression and PHQ-9 scores at baseline and each review

	Baseline (n = 270)	Review 1 (n = 234)	Review 2 (n = 196)	Review 3 (n = 167)	Review 4 (n = 144)	Review 5 (n = 113)	Total (n = 1124)
R8 Depression	38.6 (16.7)	30.3 (18.2)	27.0 (16.3)	24.9 (14.7)	22.3 (13.5)	24.6 (16.3)	29.3 (17.2)
PHQ-9	13.8 (7.7)	10.8 (7.0)	10.0 (6.6)	9.3 (6.2)	8.2 (5.8)	8.6 (6.4)	10.6 (7.1)

**Fig. 1:**
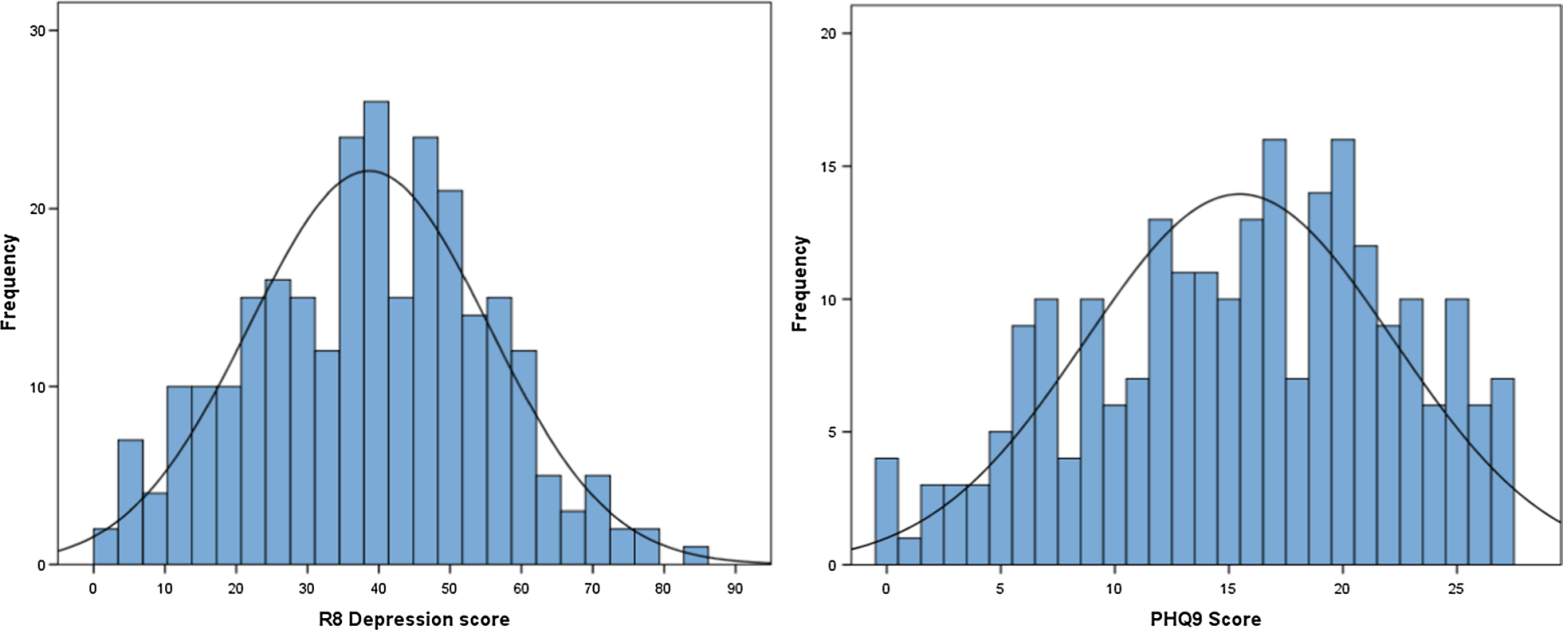
Distribution of baseline scores for **a** R8 Depression and **b** PHQ-9

**Fig. 2:**
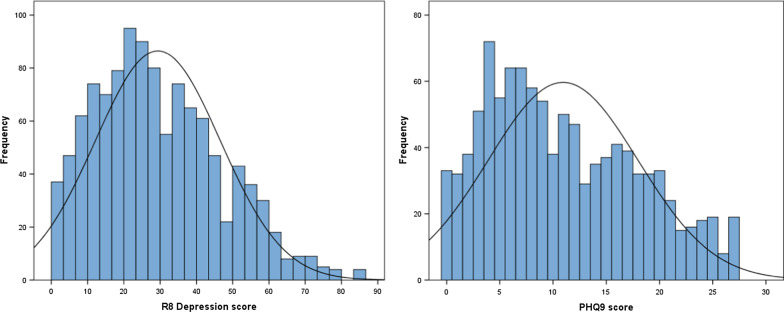
Distribution of baseline plus review scores for **a** R8 Depression and **b** PHQ-9

The distribution of the R8 Depression and PHQ-9 scores at baseline (Fig. 1) and distribution of baseline plus review scores (Fig. 2) are shown. Figure 1 shows that at baseline, the R8 Depression shows a clearer normal distribution without the ceiling effect apparent in the PHQ-9 distribution. Figure 2 shows the total clinical sample including the reviews, with a clear negative skew, expected as patients recover from their depression. The R8 Depression appears to show less of a floor effect compared to the PHQ-9.

### Internal validity

Table 4 shows the Cronbach’s alpha coefficient was 0.91 for the R8 Depression compared to 0.88 for the PHQ-9 for the baseline sample, which is high in comparison to published studies [20, 27, 28].

**Table 4:** Internal validity of R8 Depression and PHQ-9

Population	R8 Depression			PHQ-9		
	n	Cronbach’s a	Guttman	n	Cronbach’s a	Guttman
Baseline	270	0.91	0.91	236	0.88	0.85
Baseline + reviews	1124	0.92	0.92	1053	0.90	0.87
Baseline + reviews + normative	1328	0.93	0.93	1240	0.90	0.88

The Guttman’s lower bounds reliability scores, appropriate for the data distributions of interest, across the baseline plus reviews sample was high at 0.92 for the R8 Depression compared to 0.88 for the PHQ-9, as well as for the whole clinical sample (baseline plus reviews plus normative) with scores of 0.93 compared to 0.88, respectively.

Further detail relating to internal validity of the R8 Depression and differences between normative and clinical samples is included in the Additional file 1. This includes: the internal validity results for the R8 Depression and PHQ-9 separated for the normative and clinical samples; and significance testing of mean differences between the normative and combined clinical samples for total R8 Depression scores for the identified six sub-domains of the R8 Depression, and also for the total PHQ9 scores.

Further to satisfying the assumption of sampling adequacy of the whole dataset via the Kaiser–Meyer–Olkin (KMO) test (KMO = 0.906, Table 3), the results from a factor analysis performed via Principal Components Analysis [33] showed that a six factor or component solution accounts for 58.6% of the variance in the data. When Exploratory Factor Analysis was conducted with the same parameters as the PCA (Table 5), the following factors were obtained:

1. Low mood (32.7%);
2. Sleep disturbance (6.7%);
3. Low energy (5.7%);
4. Appetite and weight change (5.1%);
5. Poor cognition (4.4%), and
6. Anxiety (4.0%)

**Table 5:** Exploratory Factor Analysis of the R8 Depression using baseline sample (n = 270)

Test results			
Bartlett’s test	Chi-Square = 2897.213		
	*p* < 0.001		
Kaiser–Meyer–Olkin test	0.906		
Before rotation			
Number of factors	6		
Percentage of variance extracted	58.6%		
Scree test	1		
Direct Oblimin rotation	(Delta:.0)		
First factor		Fourth factor	
% of variance	32.7%	% of variance	5.1%
Items	Feeling worthless (0.85)	Items	Appetite change (− 0.93)
	Feeling guilty (0.62)		Weight change (− 0.63)
	Feeling hopeless (0.60)		Excessive sleep (− 0.24)
	Sadness (0.59)		
	Sensitivity to criticism (0.59)		
	Suicidal thoughts (0.53)		
	Loss of enjoyment (0.39)	Fifth factor	
	Crying (0.36)	% of variance	4.4%
	Socializing (0.36)	Items	Difficulty concentrating (0.66)
Second factor			Indecisiveness (0.56)
% of variance	6.7%		Forgetfulness (0.62)
Items	Staying asleep (0.67)		Feeling slow (0.55)
	Waking early (0.64)		
	Falling to sleep (0.37)		
	Feeling restless (0.25)	Sixth factor	
Third factor		% of variance	4.0%
% of variance	5.7%	Items	Anxiety (− 0.65)
Items	Lack of energy (0.58)		Physical symptoms (− 0.51)
	Motivation (0.53)		Health worries (− 0.39)
	Activities (0.39)		Feeling irritable (− 0.34)

Further information relating to the factor analyses is included in the Additional file 2. This includes: correlations between extracted R8 Depression factors and PHQ-9 scores for the whole sample; mean extracted R8 Depression factors and PHQ-9 scores at baseline and subsequent reviews; and the pattern matrices for factor analyses of the R8 Depression in the normative sample and baseline clinical samples.

### External validity

Table 6 shows the Pearson’s correlation coefficients between the R8 Depression and the PHQ-9 for baseline sample (0.83, *p* < 0.001), baseline plus reviews sample (0.90, *p* < 0.001), and the clinical plus normative data samples (0.91, *p* < 0.001). For the opportunistic QIDS sample (n = 193), Pearson's correlation coefficient was 0.90 between R8 Depression and PHQ-9, 0.84 between R8 Depression and QIDS, and 0.80 between PHQ-9 and QIDS. The more appropriate and rigorous Kendall’s tau coefficient was lower but still highly significant (< 0.001) at 0.76 for the entire combined clinical and normative sample.

**Table 6:** Pearson’s correlation and Kendall’s tau coefficients between the R8 Depression and the PHQ-9 scores

Sample	n	Pearson’s correlation*	*p* value**	Kendall’s tau	*p* value*
Baseline	236	0.83	< 0.001	0.64	< 0.001
Baseline + reviews	1053	0.90	< 0.001	0.75	< 0.001
Baseline + reviews + normative	1240	0.91	< 0.001	0.76	< 0.001

*r-squared, **2-tailed test

Further information relating to external validity is included in the Additional file 3. This includes: a table of correlation coefficients between total R8 Depression and PHQ-9 scores; and scatter plots between the total scores of the R8 Depression and the PHQ-9, for the normative sample, baseline clinical sample and the baseline plus review clinical sample.

### Severity categories

**Fig. 3:**
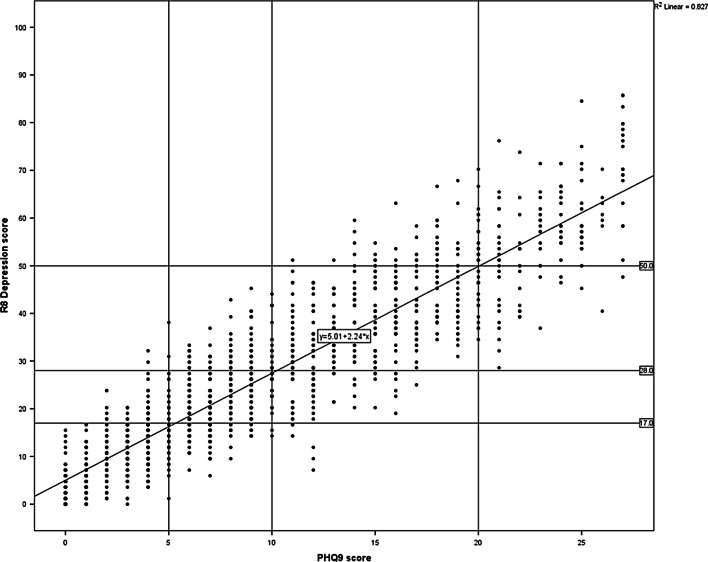
Line of best fit correlating R8 Depression and PHQ-9 total scores across combined clinical and normative samples (n = 1348)

Figure 3 shows a plot of the R8 Depression scores against the PHQ-9 scores for the whole clinical and normative datasets. This plot was used to help determine the threshold values between the four categories of “no depression”, “mild depression”, “moderate depression” and “severe depression” corresponding to the NICE treatment guidelines, as shown in Table 7.

**Table 7:** Comparison of proposed severity categories for R8 Depression to established severity categories

NICE depression severity	R8 Depression score	PHQ-9		QIDS	
		Depression severity	Score	Depression severity	Score
None	< 17	Minimal depression	0–4	None	0–5
Mild	≥ 17 and < 28	Mild	5–9	Mild	6–10
Moderate	≥ 28 and < 50	Moderate and Moderately severe	10–19	Moderate and Severe	11–20
Severe	≥ 50–100	Severe	20–27	Very severe	21–27

## Discussion

The R8 Depression was developed specifically for use as part of an automated online assessment and monitoring platform, Psynary, to assist clinicians optimising treatments for common mood and anxiety disorders. The scale was designed to cover the full range of severity presentations of depression encountered in both primary and secondary care settings. The items were informed by specialist clinical practice to encompass all the domains of depressive symptoms, and were worded to achieve optimal ease of use for patients, relevance to clinical assessments, and ease of translation to other languages. OptiMA1 has established the internal and external validation of this measure.

The parallel studies, sited in both private care and specialist public care settings helped to ensure a broad spectrum of presentations of depression and a representative clinical sample. The inclusion of baseline and review assessments captured the patient journey towards recovery and also ensured a wide distribution of severity scores.

A detailed comparison of the distributions of scores clearly highlights an important ceiling effect in the PHQ-9 (Figs. 1, 2), which limits this questionnaire’s ability to discriminate the more severe presentations of depression. Conversely, the R8 Depression approximates a normal distribution of scores more closely at baseline (Fig. 1), capturing and delineating the more severe cases of depression and not exhibiting any ceiling effects. This property is likely to be important in accurately detecting subtle changes in depression severity early during treatment.

The internal validity of the R8 Depression is excellent, exceeding that of the PHQ-9. The Principal Component Analysis together with the Exploratory Factor Analysis suggests a sub-scale structure that aligns with a clinically meaningful separation of depressive symptoms into mood, psychomotor, neuro-vegetative, cognitive and anxiety domains. This in part replicates previous factor analyses of depressive questionnaires used in STAR*D and Genome-based Therapeutic Drugs for Depression (GENDEP) clinical studies. In the latter, the psychomotor domain encompassing symptoms of interest and activity, appeared to be important in predicting poor response to antidepressant treatments [34]. Future analyses of naturalistic outcomes in the OptiMA1 will attempt to replicate these results.

A highly significant degree of external validity was demonstrated, with the Pearson correlation coefficient between the R8 Depression and the PHQ-9 with the larger clinical and normative datasets exceeding 0.90 for the entire sample. When adopting the more robust test for the non-normal distribution of interest with significant outliers, i.e. Kendall's tau method [30], the correlation coefficient is lower (0.76) but still highly significant (*p* < 0.001). The sample that included paper-based administration of the QIDS allowed for a useful triangulation to further ensure external validity, with the R8 Depression showing higher correlation with the QIDS than the correlation between the PHQ-9 and the QIDS (Pearson’s correlation coefficient = 0.84 vs 0.80).

While there has been an implicit acceptance of deviations from normality in the distribution of depression severity scores in the literature of scale validations, this paper has been explicit in its testing of assumptions of normality for the dataset. While it is reasonable to continue the tradition and widespread use of parametric analyses such as Pearson’s correlation and Cronbach’s alpha, this paper would advocate for the inclusion of more rigorous non-parametric approaches as well.

The use of large paper-based questionnaires in a clinical setting is problematic due to the constraints of time availability and complexity of calculating total scores, hence the widespread adoption of simpler tools such as the PHQ-9. The development of the R8 Depression within the automated web-based environment of Psynary obviates these constraints, enabling patients to rate their outcomes in their own time and at their own pace, away from the time-limited environment of the clinic. Importantly, this opens the opportunity of routine and detailed tests of patient-rated outcomes for depression in a clinical setting. The system captures and retains this information allowing for quantitative feedback of treatment response over time. This advantage has the potential of facilitating the creation of a framework to enable real-time routine measurement of patients’ symptoms to aid early detection of treatment response and a faster optimisation of treatment regimes.

The online Psynary platform also enables a cost-effective means of conducting clinical studies, including automation of the consent process in those jurisdictions that allow. This opens up the potential for recruiting the large clinical samples that will be needed for future clinical studies in mental health, particularly to develop strategies to personalise treatments and achieve rapid optimisation.

Having validated the R8 Depression questionnaire, the next step will be establishing the measure’s sensitivity to change. This is an essential prerequisite to extending the use of the Psynary system to aid early detection of treatment response in patients with depression.

## Conclusion

The R8 Depression has been developed as a patient-rated outcome measure for depression that is automatically administered via an online system called “Psynary”. It captures all the symptom domains of depression and, as validated in this study, it correlates well with current gold standard clinical scores, and has excellent internal and external reliability. It also has the potential for accurate measurement of early treatment response.

Future work is underway to assess the sensitivity to change and the predictive value for treatment optimisation.

### Supplementary Information

**Additional file 1: Additional analyses 3.** Tables of: the internal validity results for the R8 Depression and PHQ-9 separated for the normative and clinical samples; and significance testing of mean differences between the normative and combined clinical samples for total R8 Depression scores for the identified six sub-domains of the R8 Depression, and also for the total PHQ9 scores.

**Additional file 2: Additional analyses 1.** Further information relating to the factor analyses. Correlations between extracted R8 Depression factors and PHQ-9 scores for the whole sample; mean extracted R8 Depression factors and PHQ-9 scores at baseline and subsequent reviews; and the pattern matrices for factor analyses of the R8 Depression in the normative sample and baseline clinical samples.

**Additional file 3: Additional analyses 2.** Further information relating to external validity testing. Table of correlation coefficients between total R8 Depression and PHQ-9 scores; and scatter plots between the total scores of the R8 Depression and the PHQ-9, for the normative sample, baseline clinical sample and the baseline plus review clinical sample.

## Data Availability

The dataset supporting the conclusions of this article is available from the corresponding author on reasonable request.

## Appendix

**Table:**

R8 Depression items		
In general, over the past week…		
	1. Sadness	
		0 I don’t feel any more sad than usual
		1 I feel more sad than usual some of the time
		2 I feel sad all the time
		3 I feel so sad that I can’t stand it
	2. Loss of enjoyment	
		0 I am as interested in things and enjoy things as much as usual
		1 I am less interested or get less enjoyment from activities
		2 I have much less interest in and rarely enjoy activities
		3 I have no interest in any activities that I used to enjoy
	3. Feeling hopeless	
		0 I feel hopeful about the future
		1 Sometimes I feel I have nothing to look forward to
		2 Most of the time I feel things will get worse in the future
		3 Nearly all the time I feel totally hopeless about the future
	4. Feeling worthless	
		0 I do not feel worthless
		1 I am more self-critical than normal
		2 I feel worthless most of the time
		3 I constantly feel I am a total failure
	5. Socializing	
		0 I am able and enjoy spending time with family and friends as much as normal
		1 I don’t feel like spending time with friends as much as normal
		2 I have stopped seeing my friends and spend less time with my family
		3 I cannot stand to be around anyone and spend nearly all the time by myself
	6. Lack of energy	
		0 My energy levels are as good as normal
		1 I don’t have as much energy as normal
		2 Most days I get tired easily and everything is an effort
		3 Nearly everyday I am exhausted and cannot do anything
	7. Weight loss	
		0 I am not losing any weight
		1 I may be losing some weight
		2 I am definitely losing weight and other people notice
		3 My clothes are too big for me and other people are worrying about my weight loss
	8. Feeling guilty	
		0 I don’t feel any more guilty about things in my life than usual
		1 I feel more guilty about things some of the time
		2 Most of the time I feel very guilty about things I have done
		3 Nearly all the time I feel responsible for bad things that have happened in the world
	9. Feeling slow	
		0 My thoughts, speech and movements are not slowed down compared to normal
		1 I feel slowed down in my thoughts, speech or movements some of the time
		2 I feel slowed down most of the time, and other people have noticed
		3 I feel very slowed down nearly every day and it is very difficult to respond to what is going on around me
	10. Forgetfulness	
		0 My memory is just as good as it normally is
		1 I am more forgetful than normal
		2 I am very forgetful most of the time
		3 My memory is so bad I worry something is wrong with my brain
	11. Crying	
		0 I am not crying any more than usual
		1 I find myself crying more than usual
		2 Most of the time I am crying
		3 I feel so empty I cannot cry
	12. Weight gain	
		0 I am not gaining any weight
		1 I may be gaining some weight
		2 I am definitely gaining weight and other people notice
		3 My clothes are too tight fitting and other people are worrying about my weight gain
	13. Feeling restless and fidgety	
		0 I don’t feel restless or fidgety
		1 Sometimes I feel restless and can’t sit still
		2 Most days I feel restless and have to move around more than normal
		3 Nearly every day I have to constantly pace around and can’t stay still
	14. Health worries	
		0 I do not worry about my health any more than usual
		1 I sometimes worry more than usual about my health
		2 I constantly worry about my health
		3 I am convinced I have a serious illness or my body isn’t working properly
	15. Libido	
		0 My interest in sex is the same as normal
		1 My interest in sex is less than normal
		2 I find it very difficult to get interested in sex or enjoy myself sexually
		3 I have no interest in sex at all
	16. Anxiety	
		0 I do not feel any more anxious or tense than normal
		1 I feel more anxious or tense than normal some of the time
		2 Most of the time I feel very anxious or tense
		3 I constantly feel so anxious or tense I cannot bear it
	17. Sensitivity to criticism	
		0 I do not feel other people are particularly critical towards me
		1 I sometimes feel that other people are particularly critical towards me
		2 Most of the time I feel that other people are very critical towards me and it has affected my relationships
		3 I feel completely rejected by other people
	18. Excessive sleep	
		0 I sleep no longer than I normally do
		1 I sleep up to 2 h longer than normal, including daytime naps
		2 I sleep up to 4 h longer than normal, including daytime naps
		3 I sleep more than 4 h longer than normal, including daytime naps
	19. Activities	
		0 I have no difficulty with my usual work or leisure activities
		1 I sometimes feel weighed down or leaden
		2 Most of the time I feel weighed down or leaden, to the extent that it is difficult for me to complete my normal activities
		3 I feel paralyzed, I am unable to do any daily activities and I need support from other people
	20. Physical symptoms	
		0 I am not experiencing any increase in physical problems
		1 Sometimes I notice increased unpleasant physical symptoms, such as headaches, bowel problems, palpitations, breathing difficulties or other aches and pains
		2 Most of the time I am experiencing significant unpleasant physical symptoms
		3 I am constantly overcome by severe unbearable physical symptoms
	21. Feeling irritable	
		0 I do not get more irritable with others than normal
		1 Sometimes I get more irritable than normal
		2 Most of the time I feel irritable and easily lose my temper with others
		3 I get so angry that I have to avoid other people
	22. Suicidal thoughts	
		0 I do not think of suicide
		1 I sometimes have thoughts of killing myself
		2 I think of killing myself most of the time
		3 I constantly think of suicide, I have planned ways of killing myself or have tried to kill myself in the past week
	23. Waking early	
		0 I don’t wake up earlier than normal
		1 I wake at least 1 h earlier than normal some mornings
		2 I wake at least 1 h early more than half the mornings
		3 I wake at least 2 h early nearly every morning
	24. Motivation	
		0 I am just as motivated to do things as normal
		1 I sometimes have difficulty motivating myself to do things
		2 It is really difficult to motivate myself most of the time
		3 Nearly everyday I cannot motivate myself to do anything
	25. Increased appetite	
		0 My appetite is not increased
		1 Sometimes I feel like eating more food than normal
		2 Over half the days I eat more food than normal
		3 Nearly every day I am eating much more food than normal
	26. Staying asleep	
		0 I sleep through the night as well as I usually do
		1 I am waking briefly more often through the night than usual
		2 I am waking, and then getting back to sleep, for at least half the nights
		3 I wake for at least half an hour nearly every night
	27. Loss of appetite	
		0 My appetite is not reduced
		1 I feel like eating less food than normal
		2 I have no appetite and I have to make myself eat
		3 Most days I do not eat, and I have to really force myself to eat anything
	28. Difficulty concentrating, such as reading or watching TV	
		0 My concentration is as good as normal
		1 I have difficulty concentrating some of the time
		2 I struggle to concentrate more than half the time
		3 Nearly every day I cannot concentrate on even the simplest things
	29. Indecisiveness	
		0 I have no more problems making decisions than normal
		1 I avoid making decisions more than usual
		2 Most of the time I find it difficult to make even simple decisions
		3 I can never make any decisions
	30. Falling asleep	
		0 I have no difficulty falling asleep compared to normal
		1 It takes at least 30 min to fall asleep some nights
		2 It takes at least 30 min to fall asleep more than half the nights
		3 It takes over an hour to fall to sleep nearly every night

## Abbreviations

GENDEP: Genome-based Therapeutic Drugs for Depression
ICD-10: International Classification of Diseases 10th revision
NICE: National Institute for Health and Clinical Excellence
PHQ-9: Patient Health Questionnaire
QIDS-SR16: Quick Inventory of Depressive Symptomatology Self-Report
