# Supplementary material for: Validation of a novel online depression symptom severity rating scale: the R8 Depression
Source: Health Qual Life Outcomes. 2021 Jun 12;19:163. doi: 10.1186/s12955-020-01654-z (PMC8196428; doi:10.1186/s12955-020-01654-z)

**Additional analyses 3**

Table 1 shows the internal validity results for the R8 Depression and PHQ-9 separated for the normative and clinical samples.

**Table 1. Internal validity of R8 Depression and PHQ-9**

|  | R8 Depression | | | PHQ-9 | | |
| --- | --- | --- | --- | --- | --- | --- |
| **Population** | **n** | **Cronbach’s a** | **Guttman** | **n** | **Cronbach’s a** | **Guttman** |
| **Baseline** | 270 | 0.91 | 0.91 | 236 | 0.88 | 0.85 |
| Baseline + reviews | 1124 | 0.92 | 0.92 | 1053 | 0.90 | 0.87 |
| Baseline + reviews + normative | 1328 | 0.93 | 0.93 | 1240 | 0.90 | 0.88 |
| Normative | 204 | 0.93 | 0.93 | 187 | 0.89 | 0.90 |

Table 2 displays significance testing of mean differences between the normative and combined clinical samples for: total R8 Depression scores for the identified six sub-domains of the R8 Depression, and also for the total PHQ9 scores. Equal variance is not assumed based on the Levene test. The results below show that there is a highly significant difference between the normative and clinical samples across all these means.

**Table 2. Independent t-tests of mean R8 Depression total and sub-domain scores, and PHQ-9 total scores, between normative and combined clinical samples**


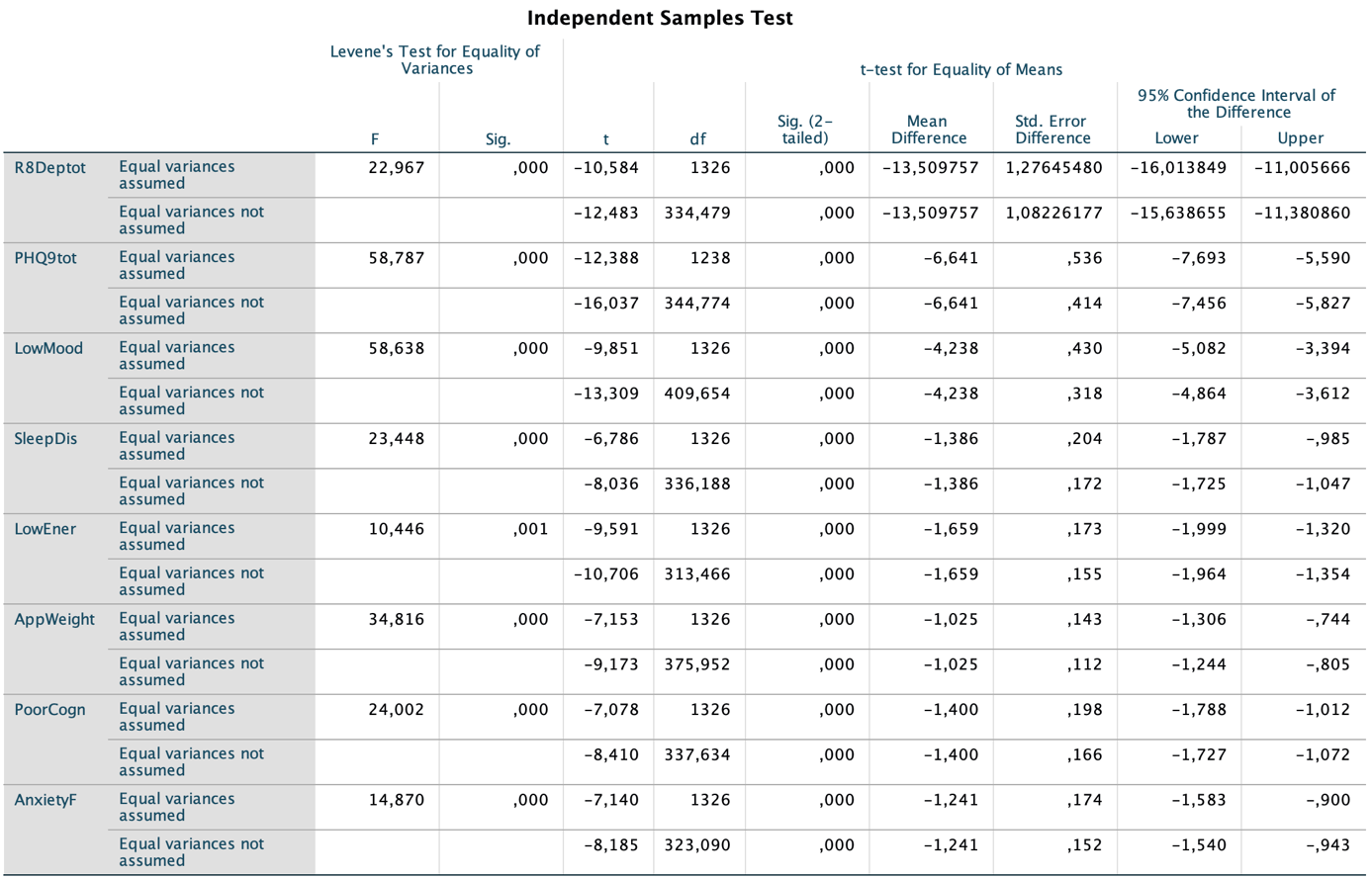

Supplement: Supplementary file 1 — Additional file 1: Additional analyses 3. Tables of: the internal validity results for the R8 Depression and PHQ-9 separated for the normative and clinical samples; and significance testing of mean differences between the normative and combined clinical samples for total R8 Depression scores for the identified six sub-domains of the R8 Depression, and also for the total PHQ9 scores. [file 12955_2020_1654_MOESM1_ESM.docx]
