# Supplementary material for: Validation of a novel online depression symptom severity rating scale: the R8 Depression
Source: Health Qual Life Outcomes. 2021 Jun 12;19:163. doi: 10.1186/s12955-020-01654-z (PMC8196428; doi:10.1186/s12955-020-01654-z)

**Additional analyses 1**

Table 1 below shows Pearson correlations of individual R8 Depression factors against the PHQ9. Given the limited symptom and hence domain coverage of the PHQ9 it is expected that some domains (e.g. low mood) show a greater level of correlation than others (e.g. atypical symptoms).

**Table 1. Pearson’s correlation and Kendall’s tau coefficients between the extracted R8 Depression factors and the PHQ-9 scores for the whole sample (N=1240)**

| R8 depression factors | Pearson’s correlation* | *p*-value** | Kendall’s tau | *p*-value* |
| --- | --- | --- | --- | --- |
| Low mood | 0.86 | <0.001 | 0.70 | <0.001 |
| Sleep disturbance | 0.54 | <0.001 | 0.42 | <0.001 |
| Low energy | 0.82 | <0.001 | 0.68 | <0.001 |
| Appetite and weight change | 0.54 | <0.001 | 0.41 | <0.001 |
| Poor cognition | 0.72 | <0.001 | 0.57 | <0.001 |
| Anxiety | 0.69 | <0.001 | 0.53 | <0.001 |

*r-squared, **2-tailed test.

Table 2 displays the mean scores and standard deviations for each of the factors of the R8 Depression over time. These results suggest there is no obvious difference in these changes over time.

**Table 2. Mean (S.D.) Extracted R8 Depression factors and PHQ-9 scores at baseline and each review**

|  | Baseline  (n=270) | Review 1  (n=234) | Review 2  (n=196) | Review 3  (n=167) | Review 4  (n=144) | Review 5  (n=113) | Total  (n=1124) |
| --- | --- | --- | --- | --- | --- | --- | --- |
| Low  mood | 11.8 (5.5) | 8.0 (6.1) | 7.1 (5.7) | 6.3 (5.0) | 5.4 (4.6) | 5.9 (5.8) | 8.0 (5.9) |
| Sleep disturbance | 4.8 (3.0) | 3.5 (2.7) | 3.2 (2.6) | 3.0 (2.1) | 2.7 (2.3) | 3.1 (2.5) | 3.5 (2.8) |
| Low  energy | 4.9 (2.0) | 3.9 (2.4) | 3.4 (2.2) | 3.2 (2.1) | 2.9 (2.1) | 3.1 (2.5) | 3.7 (2.3) |
| Appetite  and weight change | 3.0 (2.1) | 2.3 (2.0) | 2.2 (1.8) | 2.2 (2.0) | 2.1 (2.0) | 2.2 (1.8) | 2.4 (2.0) |
| Poor  cognition | 4.4 (2.6) | 3.4 (2.9) | 2.9 (2.6) | 2.7 (2.1) | 2.4 (2.3) | 2.7 (2.6) | 3.3 (2.7) |
| Anxiety | 4.9 (2.1) | 3.4 (2.4) | 3.1 (2.1) | 2.7 (2.1) | 2.3 (1.9) | 2.7 (2.2) | 3.4 (2.4) |

Tables 3 & 4 display the pattern matrices from the exploratory factor analyses for the baseline clinical and normative samples. The normative sample yields a five factor solution, which mirrors the first five factors from the baseline clinical EFA, namely low mood, sleep disturbance, psychomotor changes, appetite and weight change and cognition. The anxiety factor is lost, with these items being redistributed amongst the other factors.

**Table 3. Pattern Matrix for factor analysis of the R8 Depression in the normative sample**

**
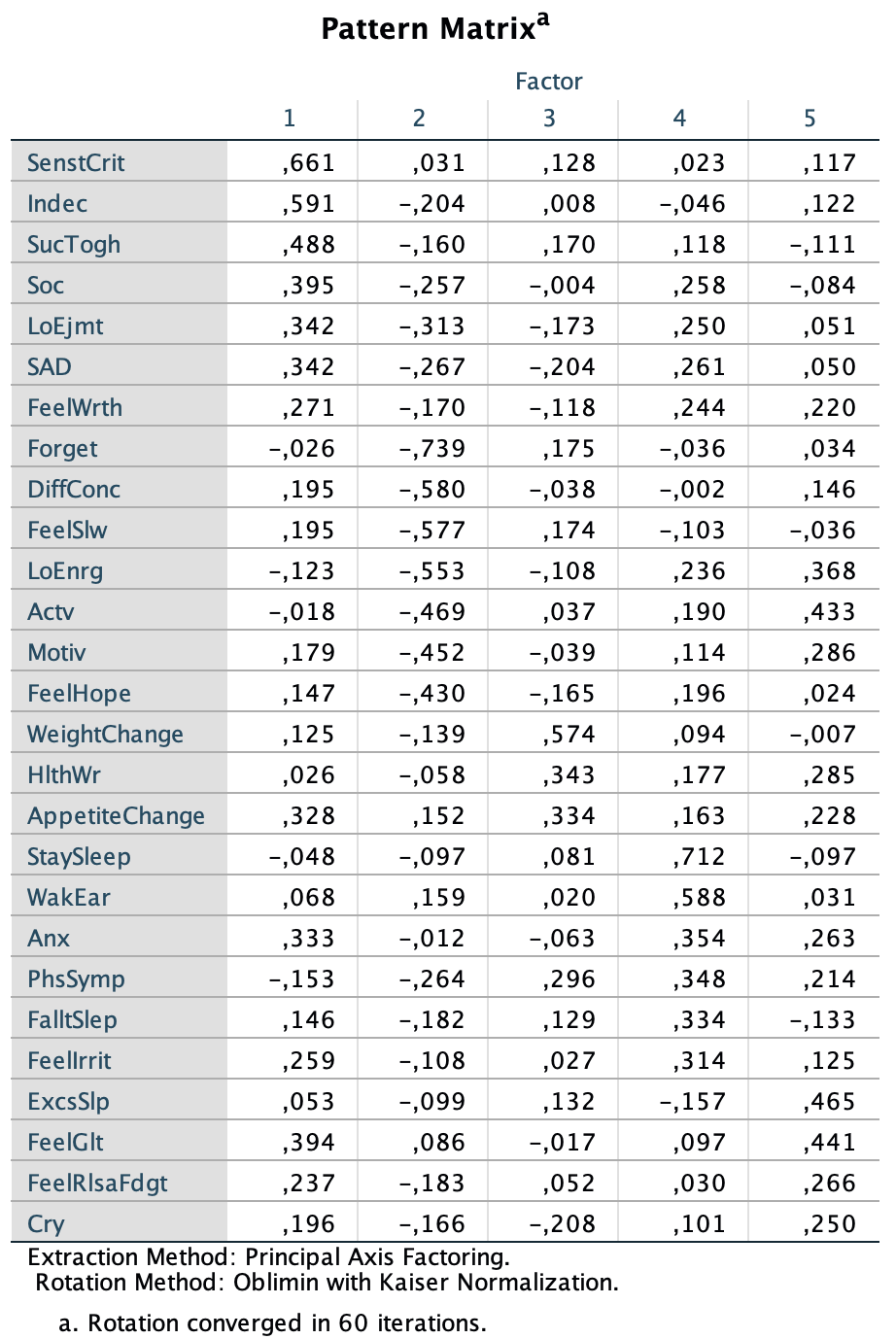
**

**Table 4. Pattern Matrix for factor analysis of the R8 Depression in the baseline clinical sample**


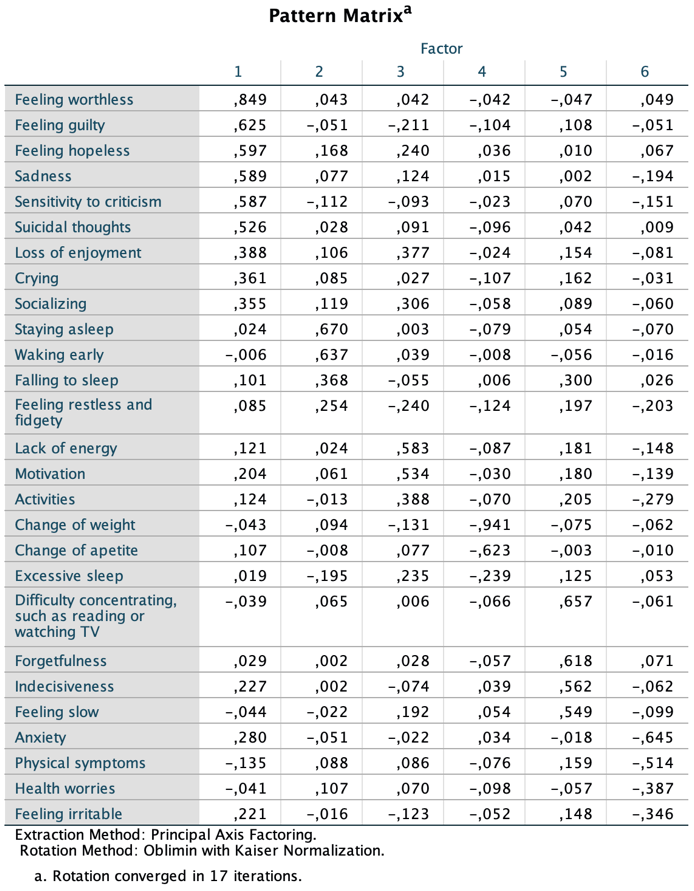

Supplement: Supplementary file 2 — Additional file 2: Additional analyses 1. Further information relating to the factor analyses. Correlations between extracted R8 Depression factors and PHQ-9 scores for the whole sample; mean extracted R8 Depression factors and PHQ-9 scores at baseline and subsequent reviews; and the pattern matrices for factor analyses of the R8 Depression in the normative sample and baseline clinical samples. [file 12955_2020_1654_MOESM2_ESM.docx]
