# Supplementary material for: Validation of a novel online depression symptom severity rating scale: the R8 Depression
Source: Health Qual Life Outcomes. 2021 Jun 12;19:163. doi: 10.1186/s12955-020-01654-z (PMC8196428; doi:10.1186/s12955-020-01654-z)

**Additional analyses 2**

Table 1 shows the correlations between the total R8 Depression and PHQ-9 scores according to the sub-groups of normative, baseline and combined clinical datasets. Due to the ceiling effect in severe depression observed for the PHQ-9, as expected, these correlations are not as strong for the baseline clinical sample.

**Table 1. Pearson’s correlation and Kendall’s tau coefficients between the R8 Depression and the PHQ-9 scores**

| Sample | n | Pearson’s correlation* | *p*-value** | Kendall’s tau | *p*-value* |
| --- | --- | --- | --- | --- | --- |
| Baseline | 236 | 0.83 | <0.001 | 0.64 | <0.001 |
| Baseline + reviews | 1053 | 0.90 | <0.001 | 0.75 | <0.001 |
| Baseline + reviews + normative | 1240 | 0.91 | <0.001 | 0.76 | <0.001 |
| Normative | 187 | 0,92 | <0.001 | 0.76 | <0.001 |

*r-squared, **2-tailed test.

Figures 1,2 & 3 display how the linear regression results between the total scores of the R8 Depression and the PHQ-9 vary between the different sub-groups, i.e. normative sample, baseline clinical sample and the baseline plus review clinical sample. This illustrates the impact of changes in clinical severity from severe depression, through stages of recovery to remission, on the linear regression results.

**Figure 1. Scatter plot of total PHQ-9 scores by total R8 Depression scores in the normative dataset only.**

**
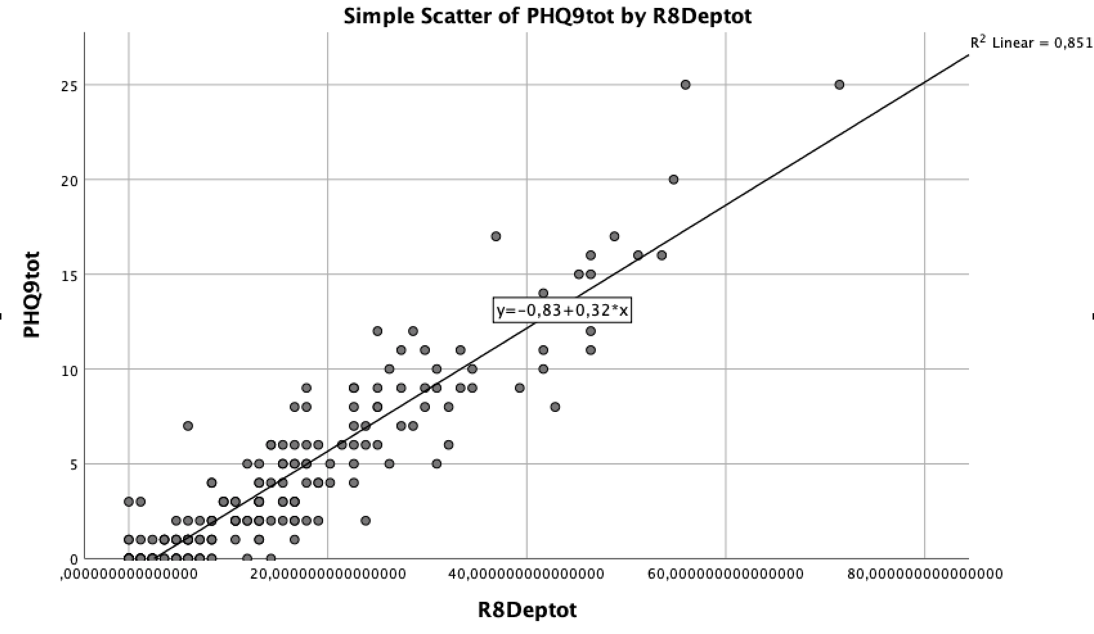
**

**Figure 2. Scatter plot of total PHQ-9 scores by total R8 Depression scores in the combined baseline and review clinical dataset.**


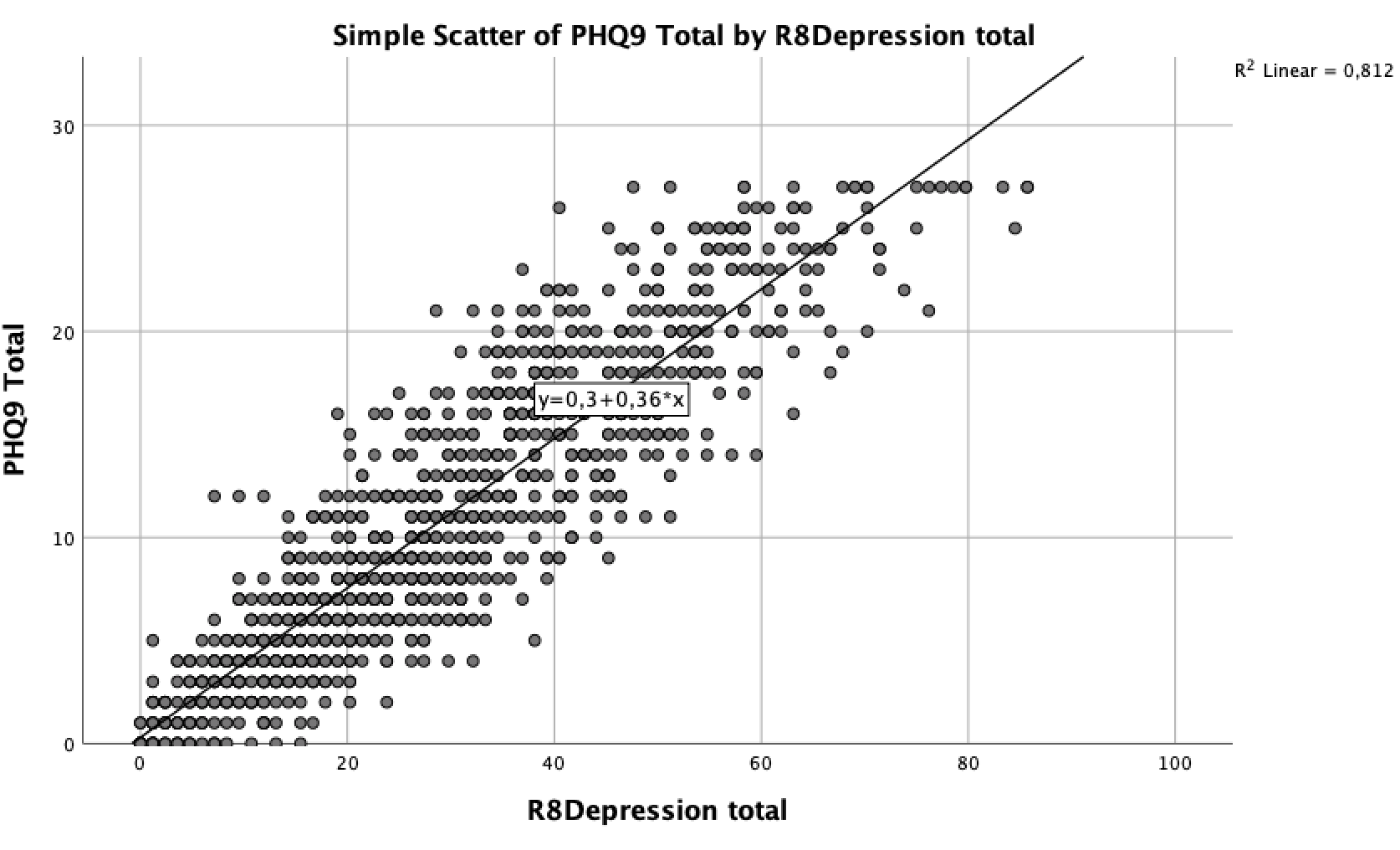


**Figure 3. Scatter plot of total PHQ-9 scores by total R8 Depression scores in the baseline clinical dataset only.**


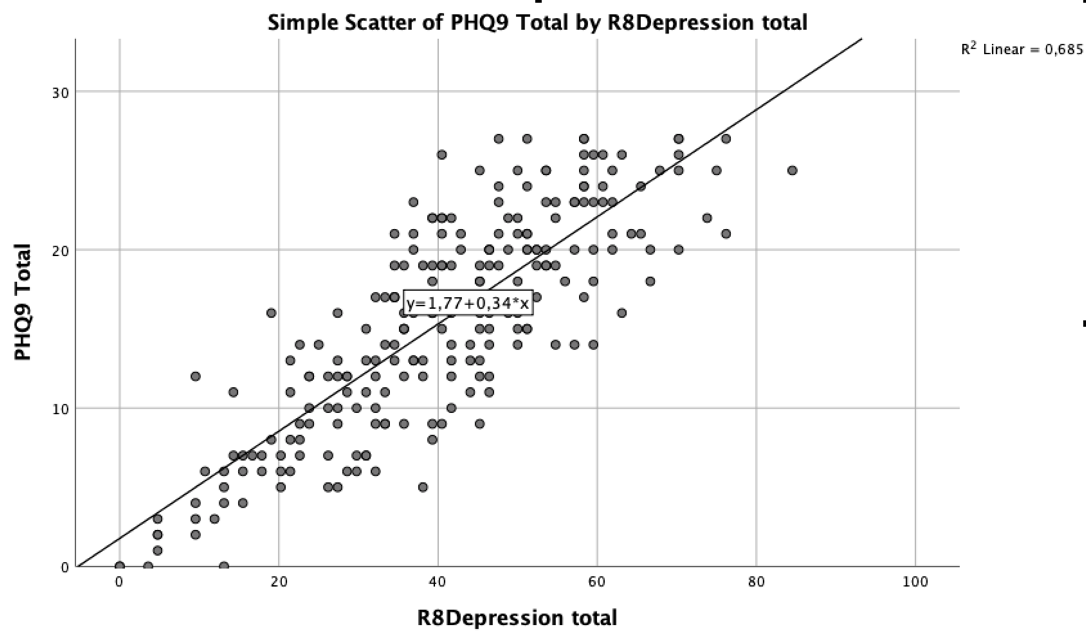

Supplement: Supplementary file 3 — Additional file 3: Additional analyses 2. Further information relating to external validity testing. Table of correlation coefficients between total R8 Depression and PHQ-9 scores; and scatter plots between the total scores of the R8 Depression and the PHQ-9, for the normative sample, baseline clinical sample and the baseline plus review clinical sample. [file 12955_2020_1654_MOESM3_ESM.docx]
